# Supplementary material for: WordCommentsAnalyzer: A windows software tool for qualitative research
Source: F1000Res. 2018 Sep 4;7:536. Originally published 2018 May 3. [Version 2] doi: 10.12688/f1000research.14819.2 (PMC6137411; doi:10.12688/f1000research.14819.2)
Supplement: Supplementary file 3 [file f1000research-7-17614-s0002.tgz › b2a79496-4ada-47c4-9682-a99ce867836d.zip!/Eight Iranian Health Journals/The Journal of Toloo-e-Behdasht_28.docx]

© The Journal of Toloo-e-Behdasht. This work is licensed under CC BY 4.0 (https://creativecommons.org/licenses/by/4.0/).

Volume & Issue: دوره 12 - شماره 4

Title: بررسی کارایی زائدات گیاه آفتابگردان در حذف کادمیوم از محلولهای آبی

http://tbj.ssu.ac.ir/article-1-1815-fa.html

مقدمه : کادمیوم از جمله آلاینده هایی است که توسط آژانس حفاظت محیط زیست آمریکا(USEPA) در زمره آلاینده های دارای اولویت قرار دارد. آلاینده های دارای اولویت، آلاینده هایی هستند که در صورت مواجهه با موجودات زنده می توانند باعث ایجاد سرطان، جهش زایی و ناقص الخلقه زایی شوند. هدف از این تحقیق بررسی تأثیر زائدات گیاه آفتابگردان در حذف کادمیوم از محلولهای  آبی بوده است. روش بررسی: این تحقیق یک مطالعه تجربی است که در مقیاس آزمایشگاهی انجام شده است. مطالعه در یک سیستم ناپیوسته انجام شده و اثر فاکتورهای زمان تماس( 5، 10، 15، 30، 45، 60 و 120دقیقه )،pH     (2، 4، 6، 7) ، غلظت کادمیوم   (15، 30 و 60 میلی گرم درلیتر ) و دوزهای جاذب (2/0، 6/0 و 1گرم در100 میلی لیتر ) بررسی شده است. و در نهایت نتایج توسط مدل های سنتیک تحلیل گردید. یافته ها : براساس نتایج، بیشترین راندمان حذف کادمیوم 95%بوده که در 5 دقیقه اول اتفاق افتاده است. pH بهینه در این تحقیق 6 به دست آمد. سنتیک جذب کادمیوم توسط زائدات گیاه آفتابگردان از مدل درجه دوم با ضریب همبستگی R2=0.999  پیروی می کند. حداکثر ظرفیت جاذب 2/20میلی گرم بر گرم بود. نتیجه گیری : نتایج حاصل از تحقیق  نشان داد که با افزایش pH  محلول از 2 به 6 کارآیی حذف  کادمیوم افزایش یافته ودر7= pH دوباره کاهش می یابد.کارایی حذف کادمیوم با افزایش جرم جاذب و زمان تماس افزایش و با افزایش  غلظت اولیه کادمیوم کاهش  می یابد.

Title: بررسی اثر مکمل کوآنزیم Q10 بر مقاومت انسولین و قند خون بیماران مبتلا به دیابت نوع دو

http://tbj.ssu.ac.ir/article-1-150-fa.html

سابقه و اهداف: دیابت نوع دو یکی از علل مهم مرگ و میر در جهان محسوب می شود و شیوع آن در سراسر دنیا در حال افزایش است. دیابت به طور عموم به عنوان یک بیماری اکسیداتیو شناخته شده است. از جمله آنتی اکسیدان هایی که امروزه در بیماری های اکسیداتیو استفاده می شود، مکمل کوآنزیم کیوتن CoQ10)) است. این مطالعه با هدف بررسی تاثیر مکمل 0 CoQ1بر مقاومت انسولینی و قند خون بیماران مبتلا به دیابت نوع دو طراحی شده است. روش بررسی : این مطالعه یک کارآزمایی بالینی تصادفی دو سوکور می باشد که با مشارکت 70 بیمار مبتلا به دیابت نوع دو تحت پوشش مرکز تحقیقات دیابت یزد در طول سال 1390 انجام گرفت. افراد به طور تصادفی به دو گروه CoQ10 و دارونما تقسیم شدند. هر دو گروه به مدت 12 هفته روزانه 2 کپسول 100 میلی گرمی محتوی CoQ10 یا دارونما مصرف کردند. شاخص های مختلف قند خون و مقاومت انسولینی قبل و بعد از مطالعه اندازه گیری شد. یافته ها : شصت و دو نفر مطالعه را به پایان رساندند. میانگین غلظت قند خون ناشتا، میانه انسولین و میانه عملکرد ترشحی سلول های بتای پانکراس بعد از مداخله در دو گروه تفاوت معنی داری پیدا نکرد. میانگین تغییرات حساسیت به انسولین پس از مداخله در گروه کوکیوتن افزایش معنی داری پیدا کرده بود (12.1+67.1در برابر -19.1+50.1: P<0.05 ). میانه تغییرات شاخص HOMA (مقاومت به انسولین) پس از مداخله میان دو گروه تفاوت معنی داری داشت (P<0.05). بحث : مطالعه حاضر نشان می دهد مصرف مکمل کوآنزیم Q10 در افزایش حساسیت به انسولین و کاهش مقاومت انسولین در بیماران مبتلا به دیابت نوع دو اثر معنی داری دارد. بنابر نتیجه این مطالعه و اثر مفید این مکمل در مطالعات مشابه؛ مصرف این مکمل در بیماران دیابتی قابل توصیه است.

Title: بررسی اثر مکمل کوآنزیم Q10 بر مقاومت به انسولین و قند خون بیماران مبتلا به دیابت نوع دو

http://tbj.ssu.ac.ir/article-1-1816-fa.html

چکیده مقدمه: دیابت نوع دو یکی از علل مهم مرگ و میر در جهان محسوب می شود و شیوع آن در سراسر دنیا در حال افزایش است. دیابت به طور عموم به عنوان یک بیماری اکسیداتیو شناخته شده است. از جمله آنتی اکسیدان هایی که امروزه در بیماری های اکسیداتیو استفاده می شود، مکمل کوآنزیم کیوتن CoQ10)) است. این مطالعه با هدف بررسی تاثیر مکمل 0 CoQ1بر مقاومت انسولینی و قند خون بیماران مبتلا به دیابت نوع دو طراحی شده است. روش بررسی : این مطالعه یک کارآزمایی بالینی تصادفی دو سوکور می باشد که با مشارکت 70 بیمار مبتلا به دیابت نوع دو تحت پوشش مرکز تحقیقات دیابت یزد در طول سال 1390 انجام گرفت. افراد به طور تصادفی به دو گروه CoQ10 و دارونما تقسیم شدند. هر دو گروه به مدت 12 هفته روزانه 2 کپسول 100 میلی گرمی محتوی CoQ10 یا دارونما مصرف کردند. شاخص های مختلف قند خون و مقاومت انسولینی قبل و بعد از مطالعه اندازه گیری شد. یافته ها: شصت و دو  نفر مطالعه را به پایان رساندند. میانگین غلظت قند خون ناشتا، میانه انسولین و میانه عملکرد ترشحی سلول های بتای پانکراس بعد از مداخله در دو گروه تفاوت معنی داری پیدا نکرد. میانگین تغییرات حساسیت به انسولین پس از مداخله در گروه کوکیوتن افزایش معنی داری پیدا کرده بود(1/67+1/12% در برابر 1/50+1/19-%  :05/0> p) میانه تغییرات شاخص HOMA (مقاومت به انسولین) پس از مداخله میان دو گروه تفاوت معنی داری داشت (05/0> p). نتیجه گیری: مطالعه حاضر نشان می دهد مصرف مکمل کوآنزیم Q10 در افزایش حساسیت به انسولین و کاهش مقاومت انسولین در بیماران مبتلا به دیابت نوع دو اثر معنی داری دارد. بنابر نتیجه این مطالعه؛ مصرف این مکمل در بیماران دیابتی  قابل توصیه است. این مقاله حاصل از پایان نامه دانشجوی کارشناسی ارشد دانشکده بهداشت، دانشگاه علوم پزشکی و خدمات  بهداشتی درمانی شهید صدوقی یزد می باشد.

Title: امکان سنجی تولید کمپوست با استفاده از پوست پسته و مخلوط پوست پسته و فضولات مرغی

http://tbj.ssu.ac.ir/article-1-1817-fa.html

مقدمه: پوست پسته یکی از محصولات فرعی فرآوری پسته است، که بعلت فسادپذیری سریع باعث آلودگی محیط زیست و ایجاد مشکلات بهداشتی شده است. لذا بایستی به طریق مناسب، بهداشتی و اقتصادی دفع گردد. هدف از این تحقیق تعیین قابلیت کمپوست شدن پوست پسته و مخلوط پوست پسته با فضولات خام مرغی با استفاده از پارامترهای رسیدگی کمپوست  است. روش بررسی: مطالعه توصیفی – مقطعی است که در فاصله زمانی مهر لغایت بهمن 1390 در شهر رفسنجان انجام شد. پوست پسته جمع آوری شده در دو ویندرو که یکی فقط پوست پسته و دیگری مخلوط پوست پسته با فضولات خام مرغی قرار داده شد. ویندرو ها در فواصل زمانی 7، 14، 21، 28، 35، 42، 70، 80، 110، 120 روز به صورت دستی زیرورو شدند و در این زمان ها نمونه برداری از آنها صورت گرفت. پارامترهای شیمیایی شامل pH ،EC ، کربن محلول در آب، نسبت C/N، بعد از هر زیرورو نمودن اندازه گیری شد. یافته ها: میانگین مقادیر پارامترهای شیمیایی رسیدگی در کمپوست پوست پسته و کمپوست مخلوط پوست پسته با فضولات مرغی به ترتیب، نسبت C/N، 14/0±71/18و 09/0±92/11، pH 03/.0±93/7 و 11/0±11/8، کربن محلول در آب (میلی گرم بر گرم) 16/0±23/7 و 29/0±81/3، به دست آمد. نتیجه گیری: مدت زمان رسیدگی کمپوست مخلوط پوست پسته با فضولات مرغی 70 روز بود. کیفیت هر دو کمپوست تولیدی در اغلب موارد در محدوده استاندارد کمپوست ایران قرار دارند و به منظور حفظ بهداشت محیط منطقه، تولید و اجتناب از هدر رفت منابع مالی پیشنهاد می گردد.

Title: کارایی زئولیت طبیعی اصلاح شده در حذف 6،4،2 تری کلروفنل از محلول‌های آبی

http://tbj.ssu.ac.ir/article-1-1818-fa.html

چکیده مقدمه: صنایع متعدی از قبیل سازندگان آفت­کش­ها، رنگ و دارو مقادیر زیادی 6،4،2- تری­کلروفنل (TCP) تولید می­نمایند. این ترکیب به دلیل سمیت بالا و آلودگی محیط زیست در گروه آلاینده­های دارای تقدم طبقه­بندی شده است. این مطالعه با هدف بررسی کارایی زئولیت (کلینوپتی­لولایت) اصلاح شده با سورفکتانت در حذف TCP از محلول­های آبی انجام شد. روش بررسی:. در این مطالعه تجربی، پس از دانه­بندی و اصلاح زئولیت با سورفکتانت هگزادسیل­تری­متیل­آمونیوم کلراید، اثر پارامترهای pH (3، 5، 7 و9)، زمان تماس (5 تا 240 دقیقه)، غلظت اولیه تری­کلروفنل (50 تا200  میلی­گرم بر لیتر)، غلظت جاذب (10 تا 60 گرم بر لیتر) در مقیاس آزمایشگاهی بصورت ناپیوسته مورد بررسی قرارگرفت. غلظت TCP در نمونه­ها با استفاده از اسپکتروفتومتر در طول موج 517 نانومتر اندازه­گیری شد. یافته ها: نتایج نشان داد که، میزان جذب TCP پس از 45 دقیقه به تعادل می­رسد و با افزایش غلظت از 50  به 200 میلی­گرم بر لیتر، میزان جذب از 04/4 میلی­گرم در گرم به 22/12 میلی­گرم در گرم افزایش می­یابد. طبق نتایج حاصل شده، میزان ظرفیت جذب TCP در    pH 3، 5، 7 و 9 به ترتیب برابر02/7، 7، 76/4 و 16/4 میلی­گرم در گرم می­باشد. افزایش دوز جاذب از 10 به 60 گرم بر لیتر در غلظت ثابت 200 میلی­گرم در لیتر TCP نیز باعث افزایش کارایی حذف از 41/ 60 درصد به 29/87 درصد و همچنین کاهش ظرفیت جذب از 78 /12 میلی­گرم در گرم به 85/2 میلی­گرم در گرم می­شود. به علاوه نتایج نشان دادکه داده­های حاصل،  از ایزوترم جذب لانگمیر (989/0R2=  ) و سینتیک درجه دوم کاذب (99/0<R2 ) تبعییت می­کنند. نتیجه­ گیری: نتایج این مطالعه نشان می­دهد زئولیت ایرانی اصلاح شده در مقایسه با زئولیت­های دیگر نقاط دنیا، قابلیت خوبی در حذف TCP دارد. با توجه به ارزان، در دسترس بودن و سادگی اصلاح، می­توان این زئولیت را جهت حذف کلروفنل­ها از آب و فاضلاب بکار برد. این مقاله حاصل از پایان نامه کارشناسی ارشد دانشگاه علوم پزشکی تهران می باشد.

Title: ارزیابی عملکرد برنامه پزشک خانواده روستایی: مطالعه موردی دانشگاه علوم پزشکی کرمان

http://tbj.ssu.ac.ir/article-1-1820-fa.html

چکیده مقدمه: بهبود مستمر عملکرد مستلزم ارزیابی پیشرفت هر برنامه، شناسایی چالشهای پیش رو و کسب بازخورد از میزان اجرای سیاستهای تدوین شده است. برنامه پزشک خانواده در ارتقای سطح سلامتی جامعه نقش مهمی دارد. این پژوهش با هدف ارزیابی عملکرد اجرای برنامه پزشک خانواده در دانشگاه علوم پزشکی کرمان انجام پذیرفت. روش بررسی :مطالعه حاضر توصیفی بود که به صورت موردی انجام پذیرفت. محیط پژوهش فعالیت های پزشک خانواده را تمامی شهرستانهای تحت پوشش دانشگاه علوم پزشکی کرمان تشکیل داد. داده ها با استفاده از فرم های آماری در نظام روتین آمار و اطلاعات، در سه محور عملکرد فنی (5 شاخص)، منابع تسهیلاتی و انسانی (6 شاخص) و منابع مالی (2 شاخص) در محدوده تحت پوشش دانشگاه علوم پزشکی کرمان جمع آوری گردید. داده های جمع آوری شده با استفاده از آمارهای توصیفی (فراوانی، درصد) و نمودارها از طریق نرم افزار  Excelمورد تجزیه و تحلیل قرار گرفت. بازه زمانی 1385 تا 1389 برای بررسی شاخصها تعیین و حداقل دوره بررسی برای آنها یک دوره 3 ساله در نظر گرفته شد. یافته ها: میانگین ارجاعات به پزشک متخصص در دوره 3 ساله 09/9 درصد بود. سهم ویزیت های دهگردشی از کل ویزیتها در همان دوره 96/9 درصد تعیین گردید. حدود 71 درصد مراکز مجری برنامه از محل زیست پزشک برخوردار بودند درحالی که این رقم با توجه به دستورالعمل برنامه باید 100 درصد باشد. در طول دوره 5 ساله برنامه میانگین ریزش پزشکان خانواده و ماماها به ترتیب 53 و 2/9 درصد بود. نتیجه گیری: علیرغم بهبود نسبی در برخی از شاخصها، میزان جذب و ریزش پزشکان در برنامه نشان داد که ماندگاری پزشکان در سطح مطلوبی قرار ندارد و با توجه به اینکه شاخص نیروی انسانی یکی از کلیدی ترین شاخص ها در بهبود عملکرد برنامه می باشد، توجه جدی را می طلبد. لذا با توجه به گسترش کمی و کیفی برنامه به مناطق شهری با جمعیت های بیشتر، نیاز به ارتقا و بهبود بویژه وجود دارد.

Title: رابطه هوش عاطفی با تعهد سازمانی مدیران بیمارستان های آموزشی شیراز، 1387

http://tbj.ssu.ac.ir/article-1-1823-fa.html

چکیده مقدمه:اخیرا هوش عاطفی به عنوان یک توانایی مهم مدیران مطرح شده است. بررسی ها نشان داده است، گوی رقابت آینده را مدیرانی خواهند برد که بتوانند به طریقی اثربخش با منابع انسانی خود ارتباط برقرار کنند. در این پژوهش، «رابطه هوش عاطفی و تعهد سازمانی مدیران ارشد و میانی بیمارستانهای آموزشی شیراز در سال 1387» بررسی شده است. روش بررسی: این پژوهش یک مطالعه توصیفی _تحلیلی مقطعی است که در سال 1387بر روی 84 مدیر ارشد و میانی که به روش نمونه گیری انتخاب شدند انجام شد. ابزار جمع آوری اطلاعات، پرسشنامه های استاندارد هوش عاطفی و تعهد سازمانی بود. داده ها با استفاده از ضریب همبستگی اسپیرمن، T.test و ANOVA تجزیه و تحلیل شده اند. یافته ها:مطالعه نشان می دهد که هوش عاطفی با تعهد سازمانی رابطه مستقیم قوی داشته و در سطح   (05/0 (P&leمعنی دار می باشد. نتایج حاصل نشان می دهد اکثر مدیران، دارای توانایی خوب هوش عاطفی بوده و تعهد سازمانی متوسط دارند نتیجه گیری:مدیران با هوش عاطفی بالا، ظرفیت چشمگیری برای پذیرش تعهد داشته و تعهد افراد به سازمان، منتج به بهره وری می شود، بنابراین مدیران باید در پرورش قابلیتهای هوش عاطفی و در نتیجه افزایش سطح تعهد سازمانی خود، کوشش نمایند. این مقاله حاصل از پایان نامه دانشجویی کارشناسی می باشد.

Title: بررسی میزان آگاهی، نگرش و عملکرد دانش‌آموزان دختر دبیرستانی در خصوص بهداشت دوران قاعدگی

http://tbj.ssu.ac.ir/article-1-1822-fa.html

مقدمه: با توجه به اینکه برنامه­ریزی برای آمادگی دختران نوجوان در روبه رو شدن با مسائل بلوغ باید بر اساس اطلاعات دقیق الگوی رفتاری و نیز نیازهای آموزشی آنها پیش­بینی و اطلاعات آموزشی لازم تعریف گردد، این مطالعه با هدف تعیین میزان اگاهی، نگرش و عملکرد دانش­آموزان دختر در خصوص بهداشت دوران قاعدگی صورت گرفته است. روش بررسی: در این مطالعه توصیفی ـ مقطعی 600 دانش­آموز دختر دبیرستان­های شهر یزد به صورت تصادفی از نواحی مختلف آموزش و پرورش شهر یزد انتخاب شدند. ابزار جمع­آوری داده­ها پرسشنامه بوده که توسط دانش­آموزان تکمیل گردیده است. داده­های جمع­آوری شده با استفاده از نرم­افزارآماری EPI 6 مورد تجزیه و تحلیل قرار گرفته است. یافته­ها: نتایج نشان می­دهد تنها 6/46 % رشد جسمی روانی اجتماعی را به عنوان مشخصات زمان بلوغ می­دانند و فقط قریب نیمی از آنان فقط قاعدگی را از تغییرات جسمی دوران بلوغ قلمداد کرده­اند. 2/12 % قاعدگی را یک بیماری دانسته و 20 % قاعده نشدن دختران را حائز اهمیت نمی­دانند. رعایت نکات بهداشتی در زمان قاعدگی در جامعه مورد مطالعه مطلوب نبوده به طوری که تنها 53% در مدت عادت ماهیانه استحمام کرده و 1/37% در این دوره پس از اجابت مزاج نظافت می­نمایند. 5/75% دختران مادر یا نزدیکان در منزل را روش کسب آموزش­های دوران بلوغ بیان نموده­اند. نتیجه­ گیری: با توجه به آگاهی و عملکرد نامناسب دختران در خصوص مسائل دوران بلوغ ضرورت ارائه برنامه­های آموزشی به ویژه مسائل بهداشتی و همچنین تصحیح باورهای غلط در مادران و دختران از طریق برگزاری کلاس­های آموزشی توسط معلمین بهداشت مدارس احساس می­شود.

Title: خصوصیات روانسنجی نسخه فارسی پرسشنامه زنان آلفا درجمعیت دانشجویی

http://tbj.ssu.ac.ir/article-1-1824-fa.html

مقدمه: شواهد جدید حاکی ازآن است که زنان پیشرفت درمحیط های کار و پست های رهبری و مدیریت را درپیش گرفته اند بنابراین برای زنانی که تمایل به شرکت درچنین نقش هایی دارند، امیدواری هایی تازه را به ارمغان آورده است. .باوجوداین، درکشور ماابزار خودگزارشی مناسبی که بتوانداینگونه زنان را(زنان آلفا )موردسنجش قراردهدوجودندارد. هدف از پژوهش حاضر بررسی کفایت شاخص های روانسنجی پرسشنامه زنان آلفادردانشجویان بود. روش بررسی: این بررسی مقطعی برروی 889 دانشجوی دختردانشگاه های پیام نورکنگاور و اسدآباد اجرا شد شرکت کنندگان با استفاده از روش نمونه گیری تصادفی نسبتی طبقه ای انتخاب و از طریق اجرای پرسشنامه زنان آلفا و خودکارآمدی برای دستیابی به ویژگی های روانسنجی پرسشنامه زنان آلفا مورد سنجش قرارگرفتند. یافته ها: با استفاده از روش تحلیل عاملی تأییدی (CFA) ساختار سه عاملی پرسشنامه زنان آلفا(رهبری، قدرت و درونگری پایین) تأیید شد و روایی همزمان بین پرسشنامه زنان آلفا با خودکارآمدی  مثبت و معنادار بود (001/0> P). علاوه بر این، نتایج پایایی و همسانی درونی  پرسشنامه با استفاده از روش آلفای کرونباخ 72/0 بود و در نهایت مولفه های به دست آمده 55/59 درصد از پراکندگی کل داده ها راتبیین می کرد. نتیجه گیری: تعداد عوامل مستخرج شده نهایی، عوامل اصلی مورد توافق  وارد،پابسن و دی پائولو را ارائه   می دهد براساس یافته های پژوهش حاضر، این پرسشنامه می تواند بعنوان ابزاری با روایی و پایایی قابل قبول جهت شناسایی زنان آلفادرداخل به کارگرفته شود.

Title: تاثیر آموزش آرامسازی بنسون بر کیفیت زندگی بیماران جراحی شده قلب باز شهر یزد

http://tbj.ssu.ac.ir/article-1-1825-fa.html

چکیده مقدمه: بیماری عروق کرونر قلب از عوامل مرگ و میر در جهان محسوب می‌شود. نتایج برخی از پژوهش‌ها نشان داده‌اند که کاربرد برخی از تکنیک‌های مدیریت استرس از طریق کاهش فشار خون، توان انقباضی عروق را کاهش می‌دهد و موجب بهبود عملکرد قلب و ارتقاء کیفیت زندگی بیماران می‌شود. این مطالعه نیز با هدف تعیین میزان تاثیر آموزش آرامسازی بنسون بر کیفیت زندگی بیماران جراحی شده قلب باز شهر یزد انجام شد. روش بررسی: این مطالعه تجربی بود و در میان 120 نفر از بیماران جراحی شده قلب باز مراجعه کننده به مرکز قلب بیمارستان افشار شهر یزد که حداقل 2 ماه از عمل جراحی آن‌ها گذشته بود انجام گرفت. تعداد 60 نفر درگروه آزمون و 60 نفر درگروه غیر آزمون جای گرفتند. ابزار جمع آوری داده‌ها پرسشنامه استاندارد کیفیت زندگی SF36 بود. داده‌های جمع آوری شده با استفاده از نرم افزار آماری SPSS-14 مورد تجزیه و تحلیل قرار گرفت. یافته‌ها: میانگین سن بیماران 44/9±95/56 سال بود که 8/65 درصد مرد بودند. میانگین نمره تمام ابعاد کیفیت زندگی بیماران در گروه آزمون بعد از مداخله آموزشی معنادار بود.به طوریکه میانگین 8 بعد کیفیت زندگی     ( بعد عملکرد فیزیکی، بعد محدودیت نقش بعلت مشکلات جسمانی، بعد محدودیت نقش بعلت مشکلات روحی، بعد نشاط، بعد سلامت روان ،بعد عملکرداجتماعی، بعد درد بدنی و بعد سلامت عمومی)پس از مداخله در گروه آزمون بهبود یافت . آزمون آماری pair T‐test نشان داد که اختلاف آماری معنی داری بین ابعاد بعد محدودیت نقش بعلت مشکلات جسمانی، محدودیت نقش بعلت مشکلات روحی،بعد نشاط،بعدسلامت روان و بعد درد بدنی در گروه غیر آزمون قبل و 2 ماه بعد از مداخله وجود نداشت. نتیجه گیری: کاربرد آموزش آرامسازی به روش بنسون موجب ارتقاء کیفیت زندگی بیماران جراحی شده قلب باز گردید. آموزش مدیریت استرس به عنوان یک مداخله روانشناسی توانسته است بر همه ابعاد کیفیت زندگی اعم از جسمانی و روانشناختی بیماران گروه آزمون تاثیرات زیاد و معنی داری نسبت به گروه کنترل داشته باشد. این امر تاثیر شگرف  آموزش مدیریت استرس در جهت بهبود مسائل روانشناختی بیماران پس از جراحی قلب     می باشد . این مقاله حاصل از پایان نامه دانشجوی کارشناسی ارشد دانشکده بهداشت، دانشگاه علوم پزشکی و خدمات  بهداشتی درمانی شهید صدوقی یزد می باشد.

Title: بررسی بیوآئروسل ها در هوای معابر شهری و روستایی دامغان در زمستان 1390

http://tbj.ssu.ac.ir/article-1-1826-fa.html

چکیده مقدمه: قارچ ها در سراسر کره زمین وجود دارند و تقریباً به اندازه گیاهان گلدار فراوان وگوناگون می باشند و به طریق اسپور زایی تولید مثل می کنند. قارچ ها قادرند در دمای بدن  رشد کرده و به راحتی افراد حساس را آلوده نموده و عفونت هایی را  ایجاد نمایند. لذا بررسی اسپورهای قارچی که یکی از شاخص های مهم کیفیت بیولوژیکی هوا  می باشد، هدف این مطالعه نیز قرار گرفته است.    روش بررسی: این پژوهش یک  مطالعه توصیفی - مقطعی می باشد که در شهر دامغان و دو روستا از توابع آن انجام گرفت. نمونه برداری با استفاده از دستگاه نمونه بردار بیوآئروسل در ارتفاع تراز تنفسی (m 2-1) به صورت نمونه برداری مستقیم روی محیط کشت سابوردکستروز آگار انجام شد. در 5 نقطه از معابر شهری و 5 نقطه از هر یک از روستا ها نمونه برداری با 2 بار تکرار  در روزهای مختلف از یک فصل و با دبی 28 لیتر بر دقیقه به مدت 5 دقیقه انجام شد. یافته ها: در این مطالعه در مجموع 132 کلنی در نمونه ها مشاهده شد، که نمونه های شهر دامغان با 64 کلنی بیشترین تعداد  و روستاهای مهماندوست با 36 و جزن با 32 ، کمترین تعدادکلنی را داشتند. بیشترین گونه های قارچی در هوای دامغان شامل؛ آسپرژیلوس فومیگاتوس، آلترناریا، پنی سیلیوم و آسپرژیلوس فلاووس بودند و بیشترین تعداد گونه های هوای روستاهای جزن و مهماندوست شامل؛ آسپرژیلوس فومیگاتوس، آسپرژیلوس فلاووس و پنی سیلیوم بود. نتیجه گیری : براساس نتایج، هوای روستاهای دامغان به لحاظ وجود اسپورهای قارچی از کیفیت مطلوبتری برخودارمی باشند. لذا حضور افراد بیمار یا حساس از این نظردر مناطق روستایی یا مناطق کم تراکم وکم تردد مشابه روستا، می تواند در ارتقای سلامتی این افراد تاثیر گذار باشد.

Title: بررسی ارتباط بین سازه های الگوی اعتقاد بهداشتی و قصد انتخاب روش زایمان

http://tbj.ssu.ac.ir/article-1-1827-fa.html

چکیده مقدمه:زایمان بعنوان یک تجربه بحرانی در دوران زندگی زنان تلقی می شودکه دارای ابعاد جسمی،اجتماعی، فیزیولوژیک،فرهنگی و روانی می باشد. تامین، حفظ و ارتقاء سلامت مادران و نوزادان بعنوان دو گروه آسیب پذیر جامعه اهمیت ویژه ای دارد. از این رو ترویج زایمان طبیعی در تمام سطوح نظام بهداشتی و درمانی کشور بعنوان سیاستی اساسی همواره مورد توجه قرار گرفته است. روش بررسی:مطالعه حاضر توصیفی بود که برروی 130نفر اززنان باردارنخست زایی که درسه ماهه سوم بارداری به مراکزبهداشتی درمانی شهر یزد مراجعه کرده و به روش نمونه گیری تصادفی انتخاب شدند،در سال 1392 انجام شده است. داده ها با استفاده ازپرسشنامه محقق ساخته که دارای 4 بخش بود وروایی وپایایی آن مورد تاییدقرارگرفته بود جمع آوری گردید. داده های گردآوری شده با استفاده از نرم افزار آماری spss نسخه  18 و با استفاده از شاخصهای میانگین و انحراف معیار توصیف شد .رابطه بین سازه ها و متغیرهای دموگرافیک  با استفاده از آزمونهای من ویتنی،کروسکال والیس و کای اسکوئر آنالیز شد. یافته ها: در این پژوهش میانگین سنی زنان73/3 ±36/24 و میانگین سن بارداری 35/2± 53/31 بود. از کل زنان باردار تحت مطالعه 2/26%  قصد انجام زایمان به روش سزارین و 8/73%  قصد انجام زایمان طبیعی را داشتند. بین قصد انتخاب روش زایمان و سازه های منافع درک شده (001/0=p )، موانع درک شد(001/0=p )، خودکارامدی درک شده(001/0=p ) و حساسیت درک شده (002/0=p )ارتباط معنی دار وجود داشت اما بین قصد انتخاب روش زایمان و سازه شدت درک شده (505/0=p )و آگاهی (756/0=p )ارتباط معناداری وجود نداشت. نتیجه گیری: نتایج نشان داد که مدل اعتقاد بهداشتی می تواند برقصد مادران تاثیر بگذارد ،لازم است برنامه آموزشی مناسب براساس یافته های بررسی تنظیم وبه زنان ارائه شود.

Title: مقایسه تاثیر دستگاه الکترولیز تجاری حاوی با الکتروهای آهن و آلومینیوم با الکترودهای زغالی بر کیفیت فیزیکو شیمیایی آب آشامیدنی

http://tbj.ssu.ac.ir/article-1-1828-fa.html

مقدمه: فروشندگان دستگاههای تصفیه آب در نقطه مصرف (خانگی) برای بازاریابی و افزایش فروش محصولات خود، با استفاده از الکترودهای تجاری آهن و آلومینیوم، کیفیت آب شرب را دستخوش تغییراتی نموده که باعث افزایش جامدات محلول و معلق آب می شود که با نصب تجهیزات تصفیه آب، آب خروجی زلالی تولید می شود. با توجه به عدم وجود دلایل علمی موجه در ایجاد لجن و تغییر رنگ آب ناشی از الکترولیز با الکترود تجاری، این مطالعه با هدف مقایسه کیفیت فیزیکو شیمیایی آب پس از الکترولیز با الکترودهای تجاری و الکترود زغالی(به عنوان شاهد) انجام شد. روش بررسی: پس از انجام فرایند الکترولیز با الکترودهای تجاری (آهن و آلومینیم بطور توام)، هریک بطور مجزا و الکترود زغالی (به عنوان شاهد) بر روی نمونه­های آب، پارامترهای کیفی سختی، قلیائیت، pH ، EC،TDS  قبل و بعد از الکترولیز به روش استاندارد مورد آزمایش قرار گرفت. پس از آنالیز داده ها، میانگین پارامترهای کیفی آب ناشی از الکترولیز الکترودهای مختلف با هم مقایسه شد. یافته­ها: pH نمونه­ها پس الکترولیز با الکترود تجاری و الکترودهای آهن-آهن افزایش معنی داری داشت(05/0p <) ولی با الکترودهای آلومینیوم- آلومینیوم تغییر معنی داری مشاهده نشد(05/0p >). سختی کل، کلسیم و منیزیم و قلیاییت نیز پس از الکترولیز باالکترود تجاری و الکترودهای آهن-آهن افزایش معنی داری داشت (05/0p <) ولی برای الکترودهای آلومینیوم - آلومینیوم بدون تغییر ماند. هدایت الکتریکی و کل جامدات محلول پس از الکترولیز با انواع الکترودها از نظر آماری بطور قابل ملاحظه ای افزایش یافته بود. رنگ نمونه ها پس از استفاده از الکترودهای آهن-آهن  و الکترود تجاری قرمز مایل به قهوه­ای و برای الکترودهای آلومینیوم سفید کدر شده در حالی که پس از استفاده از الکترود زغالی تغییری پیدا نکرده بود. نتیجه گیری: الکترولیز آب با الکترودهای تجاری مورد مطالعه باعث تولید رنگ و لجن در اثر آزادسازی یونهای آلومینیوم و آهن شده و منجر به تولید آبی کدر و سیاه می شود که به تصور اشتباه، ناخالصی های آب می باشد در حالی که رسوبات تشکیل شده ناشی از واکنش یونهای آزاد شده از الکترودها با هیدروکسید آب مورد آزمایش می باشد. پس این گونه الکترودها تقلبی بوده و استفاده های سودجویانه از آن می شود که مراکز بهداشت بایستی نسبت به افزایش آگاهی عموم و جمع آوری آنها از بازار اقدام نمایند.

Title: بررسی آگاهی، نگرش و عملکرد افراد 15 ساله و بالاتر شهر کرج در خصوص مصرف آدامس و اثرات بهداشتی آن

http://tbj.ssu.ac.ir/article-1-1829-fa.html

مقدمه: آدامس شایع‌ترین جویدنی دنیاست که اثرات مطلوب بهداشتی فراوانی همچون خنثی کردن باکتری‌های دندانی و جلوگیری از خشکی دهان به همراه دارد. با توجه به فقدان مطالعات در زمینه آگاهی افراد جامعه در مورد اثرات بهداشتی جویدن آدامس، پژوهش حاضر با هدف تعیین میزان آگاهی، نگرش و عملکرد افراد 15 ساله و بالاتر شهر کرج در این زمینه صورت گرفت. روش بررسی: در این مطالعه‌ توصیفی تعداد 400 نفر افراد 15 ساله و بالاتر شهر کرج بصورت نمونه گیری تصادفی خوشه‌ای انتخاب و مورد مطالعه قرار گرفتند. ابزار گردآوری اطلاعات پرسشنامه محقق ساخته دارای روایی و پایایی معتبر در چهار بخش: دموگرافیک 6 سوال، آگاهی 23 سوال، نگرش 7 گویه و عملکرد 7 سوال بود و به صورت مصاحبه تکمیل شد. داده‌ها توسط نرم افزار آماری SPSS و با کمک آزمون های توصیفی و تحلیلی ANOVA، T-test و ضریب همبستگی پیرسون مورد تجزیه و تحلیل قرار گرفت. یافته‌ها:  بر اساس نتایج 5/78 درصد از واحدهای پژوهش آدامس می جویدند که از این تعداد، 3/8 درصد میزان مصرف خود را هر روز اعلام کردند. ازاین تعداد تنها 7/37 درصد به علت اثرات بهداشتی آن آدامس می جویدند. بین آگاهی و سطح تحصیلات (002/0=P) و میزان مصرف آدامس (045/0=P)رابطه آماری معنی داری موجود بود. نگرش افرادی که آدامس مصرف می کردند بطور معناداری مطلوب تر از نگرش افرادی که آدامس مصرف نمی کردند بود(013/0=P). وضعیت اقتصادی (026/0=P)، سطح تحصیلات (024/0=P)، وضعیت استعمال سیگار (001/0>P) و میزان مصرف آدامس نیز (07/0=P) رابطه آماری معنی داری با وضعیت نگرش افراد داشت. بین عملکرد افراد و متغیرهای زمینه ای ارتباطی دیده نشد. بعلاوه، میانگین سنی افرادی که آدامس می جویدند بطور معنی داری (001/0>P) کمتر از افرادی بود که آدامس نمی جویدند و نیز آدامس جویدن در بین مجردین (001/0>P) و دانشجویان (002/0=P) بطور معناداری بیشتر دیده شد. نتیجه گیری: آگاهی، نگرش و عملکرد در افراد مورد مطالعه در رابطه با اثرات بهداشتی جویدن آدامس در سطح متوسطی بود، پیشنهاد می گردد از نتایج این مطالعه در جهت ارائه برنامه های آموزشی مؤثر و دقیق درمورد افزایش اثرات مثبت جویدن آدامس استفاده شود.

Title: ارزیابی کیفیت فیزیکی وشیمیایی آبهای زیرزمینی قابل شرب مناطق روستائی شهرستان خواف

http://tbj.ssu.ac.ir/article-1-1830-fa.html

مقدمه: آب از منابع محدود و طبیعی می باشد  که برای بقای انسان ضروری است. در بین منابع آبی، آبهای زیرزمینی برای انواع فعالیتهای انسانی جزء منابع ارزشمند محسوب می شوند. این تحقیق به منظور پایش کیفی پارامترهای فیزیکی و شیمیایی منابع آب شرب روستاهای شهرستان خواف  طی سال 89-88 مورد بررسی قرار گرفته است روش بررسی:مطالعه مورد نظر از نوع توصیفی ، مقطعی بوده است، نمونه برداری در فاصله  سالهای 89 – 88  به تعداد60 نمونه در دو فصل پاییز و بهار از 30 منبع آبی برداشت شده و 14 پارامتر بر اساس استاندارد متد مورد آنالیز قرار گرفتند و  با استاندارد آب ایران و سازمان حفاظت محیط زیست به لحاظ رعایت استاندارد مورد مقایسه قرار گرفتند . یافته ها:نتایج تحقیق حاصل نشان می دهد که پارامترهای سولفات، کلرور،سدیم ،هدایت الکتریکی ،کدورت به ترتیب 20،10،36،23 و 10 درصد بیشتر از حد استاندارد بوده اند.درمورد نیترات3/93 در صد در محدوده مطلوب  و 6/6 درصد بیشتر از حد استاندارد بودند ، همچنین میزان فلوئور در محدوده 9/3 - 15/0 میلی گرم در لیتر بوده که  3/33 و 10 درصد به ترتیب کمتر و بیشتر از حد استاندارد و  6/56 درصد روستاها در محدوده مطلوب قرار دارند. نتیجه گیری: در مجموع می توان نتیجه گیری نمود که پارامترهای اندازه گیری شده از جمله نیترات،  فلوراید، سولفات، کلرور،سختی،که برای سلامتی حائز اهمیت هستند در رنج  مطلوبی قرار ندارند. بنابراین دولت استراتژی خود را بر سه هدف تصفیه، بهسازی و انتقال آب شرب مناسب متمرکز نماید.

Title: کاربرد استخوان ماهی مرکب به عنوان جاذب طبیعی در حذف فلوئور مازاد از آب آشامیدنی

http://tbj.ssu.ac.ir/article-1-1831-fa.html

مقدمه: کنترل غلظت فلوئور درآب آشامیدنی به دلیل اثر بر سلامت انسان بسیار مهم است. هدف مطالعه بررسی کارآیی استخوان ماهی مرکب در حذف فلوئور مازاد از آب و تعیین تأثیر pH، غلظت جاذب، زمان تماس و غلظت اولیه فلوئور بود.  روش بررسی: مطالعه با استفاده از راکتورهای ناپیوسته در مقیاس آزمایشگاهی  انجام شد. خرد کردن و دانه بندی جاذب با استفاده از آسیاب برقی و الک های استاندارد ASTM انجام گرفت. مشخصات ساختاری وسطح ویژه جاذب با تکنیک های پراکنش پرتو ایکس(XRD)، میکروسکوپ الکترونی روبشی(SEM) و ایزوترم BET تعیین شد. غلظت فلوئور با روش نور سنجی در طول موج 570 نانومتر  تعیین شد. ایزوترم جذب و ظرفیت جاذب با مدلهای جذب فروندلیچ، لانگمیر و تمکین تعیین گردید. یافته ها: سطح ویژه جاذب 36/5 متر مربع در هر گرم و ترکیب اصلی آن کربنات کلسیم است. در  pH خنثی، دوز جاذب g/L 15، غلظت اولیه فلوئور mg/L 7 و زمان تماس 120 دقیقه راندمان حذف معادل 02/52%  بود. با افزایش دوز جاذب از  g/L 15 به g/L 45 این میزان به 2/75% افزایش یافت. داده های جذب با ایزوترم جذب لانگمیر(997/0 =R2) مطابقت دارد. حد اکثر ظرفیت جذب بر اساس مدل لانگمیر mg/g 64/1، b معادلL/mg  063/0  و  ضریب بدون بعد RL  معادل76/0 تعیین شد. بر اساس مدل فروندلیچ نیز میزان ضرائب n و k به ترتیب معادل 9/0 و 08/0 محاسبه شد.  نتیجه گیری: با افزایش زمان تماس، غلظت اولیه فلوئور، دوز جاذب و کاهش pH محلول میزان حذف افزایش می یابد.

Title: بررسی کارایی آلفا و گاما آلومینا در حذف فلوراید از آب آشامیدنی

http://tbj.ssu.ac.ir/article-1-1833-fa.html

چکیده مقدمه: فلوراید یکی از عناصر ضروری برای بدن بوده و وجود مقادیر مطلوب آن از بروز پوسیدگی دندان جلوگیری می کند. با اینحال جذب  بیش از حد فلوئور به بدن انسان  می تواند پیامدهای مزمن زیادی،  نظیر فلوئورزیس را به همراه داشته باشد. هدف از این تحقیق بررسی تأثیر آلفا و گاما آلومینا در حذف فلوراید از آب بوده است. روش بررسی: این تحقیق یک مطالعه تجربی است که در مقیاس آزمایشگاهی انجام شده است . مطالعه روی182 نمونه سنتتیک در یک سیستم ناپیوسته انجام شده و اثر فاکتورهای زمان تماس( 5 ،10، 15، 30، 45،60 دقیقه )،pH  (6،7،8)، غلظت فلوراید   (1،2،3،4،5،6 میلی گرم درلیتر ) و دوزهای جاذب (1 ، 5/2 ، 5 ، 5/7  میلی گرم درلیتر ) بررسی شده است. و در نهایت میزان مطابقت داده ها  با ایزوترم های جذب  لانگمیر و فروند لیچ تعیین گردید. یافته ها : براساس نتایج آلفا آلومینا دارای کارایی بهتری نسبت به گاما آلومینا در حذف فلوراید بوده است. به نحوی که در pH 6، دوز جاذب 5گرم در لیتر ، غلظت فلوراید 5 میلی گرم در لیتر و زمان 30 دقیقه، راندمان حذف فلوراید توسط آلفا آلومینا 85 درصد و در گاما آلومینا 4/72 درصد بوده است. بررسی ایزوترم های جذب نشان داد که جذب فلوراید توسط آلفا و گاما آلومینا  از مدل لانگمیر پیروی می کند. نتیجه گیری : نتایج حاصل از تحقیق  نشان داد که با افزایش pH  محلول کارآیی جذب  فلوراید کاهش یافته و pH بهینه در محدوده 6 تا 7 می باشد. کارایی حذف فلوراید با افزایش جرم آلومینا افزایش و با افزایش  غلظت اولیه فلوراید کاهش  می یابد و حداکثر جذب در 15 دقیقه اول واکنش حاصل می گردد. این مقاله حاصل از پایان نامه دانشجوی کارشناسی ارشد مرکز زیست فناوری پردیس بین الملل یزد می باشد.

Title: جداسازی و تشخیص مولکولی یک گونه باکتری تجزیه کننده نرمال هگزادکان از کمپوست و بررسی کارایی آن در حذف هگزادکان

http://tbj.ssu.ac.ir/article-1-1832-fa.html

مقدمه:یکی از متداول‌ترین آلاینده‌های محیط زیست، هیدروکربن‌های نفتی می‌باشد. هم‌اکنون یکی از مهم‌ترین و گسترده‌ترین چالش‌های زیست‌محیطی ایران، آلودگی خاک و آبهای زیرزمینی به گازوییل نشت یافته از جایگاه‌های پخش فرآورده‌های نفتی است.هدف از این مقاله جداسازی و شناسایی مولکولی یک باکتری تجزیه‌کننده هگزادکان از کمپوست بوده است. روش بررسی: در این پژوهش تجربی –آزمایشگاهی نرمال هگزادکان (C16H34) به عنوان آلاینده‌ی مدل هیدروکربن‌های گازوییل انتخاب گردید. سپس یک باکتری جدید تجزیه‌کننده‌ی نرمال هگزادکان از کمپوست جداسازی و خالص‌سازی شد. سپس با  انجام PCR با روش 16SrDNA باکتری مورد نظر شناسایی شد و توانایی باکتری جهت حذف هگزادکان از محیط معدنی مورد بررسی قرار گرفت.در پایان پاسخ باکتری به غلظت‌های مختلف شوری (0، 1، 5/2 و 5 درصد) بررسی شد. یافته‌ها: بر اساس مورفولوژی، آزمایش‌های بیوشیمیایی و انجام PCR با روش 16SrDNA این باکتری به نام سراتیا مارسسنس شناسایی شد. پس از 33 روز در دمای 30 درجه‌ی سلسیوس، 5/63 درصد از هگزادکان توسط باکتری مورد مصرف قرار گرفت و میزان هگزادکان از 90 میلی‌گرم به 84/32 گرم رسید. همچنین نتایج نشان داد که باکتری مورد نظر در برابر شوری مقاومت بالایی دارد و می‌تواند در غلظت 5 درصد نمک نیز رشد کند. نتیجه‌گیری:این پژوهش نشان می‌دهد که در شرایط آب و هوایی گرم و خاک نسبتا شور ایران می‌توان از باکتری سراتیا مارسسنس جهت حذف ترکیبات نفتی به‌ویژه گازوییل استفاده کرد. به دلیل رشد این باکتری در شوری بالا، نتیجه گرفته شد که این باکتری مقاوم به شوری یا هالوتولرانت می‌باشد.

Title: تحلیل حساسیت نسبت به اریبهای پنهان در کارآزمائی های بالینی کنترل نشده و کاربرد آن در مقایسه تاثیر دو رژیم درمانی بیماران مبتلا به کبد چرب غیر الکلی

http://tbj.ssu.ac.ir/article-1-1834-fa.html

چکیده مقدمه: کارآزمائیهای بالینی تصادفی شده (Rct ) به عنوان استاندارد طلایی در بررسی تاثیر روشهای درمانی و پیشگیری از بیماریها محسوب می شوند و ویژگی مهم آنها تخصیص تصادفی بین گروههای مورد مطالعه می باشد. در مطالعاتی که تخصیص تصادفی بین گروههای مورد مقایسه وجود ندارد اریبهای پنهان ممکن است منجر به تحریف نتایج گردد. تحلیل حساسیت روش مناسبی برای بررسی اثرات اریبهای پنهان می باشد. در این مطالعه به معرفی و کاربرد این روش می پردازیم و سپس کاربرد آن را در یک کارآزمایی بدون تخصیص تصادفی نشان می دهیم. روش بررسی: به منظور انجام تحلیل حساسیت از روش ارائه شده توسط آقای Resenbum استفاده گردید و با برنامه نویسی در محیط نرم افزار R نتایج تحلیل شدند. داده های مورد استفاده در این روش مربوط به یک مطالعه کارآزمائی بالینی تصادفی نشده می باشد که رژیم درمانی با و بدون ورزش را در درمان کبد چرب غیر الکلی مورد مقایسه قرار داده است. جامعه مورد پژوهشی بیماران مبتلا به کبد چرب غیر الکلی که با سونوگرافی تشخیص داده شده اند،می باشد. یافته ها: در تحلیل نتایج به آزمونهای معمول آماری تفاوت معنی داری بین مقدار آنزیم های کبدی ALT و AST و ALK وجود داشت. ولی مدل تحلیل حساسیت نشان داد که نتایج نسبت به اریبهای پنهان حساس بودند و این اریبها منجر به ایجاد انحراف درنتایج مطالعه گردید. نتیجه گیری: نتایج مدل تحلیل حساسیت نشان می دهد که مصرف ویتامین E بر روی مقدار آنزیم های کبدی تاثیری ندارد. در این مطالعه حساسیت به اریبهای پنهان پایین می باشد و این اریبها نتایج مطالعه را تحت تاثیر قرار داده اند. این مقاله حاصل از پایان نامه دانشجوی کارشناسی ارشد دانشکده بهداشت، دانشگاه علوم پزشکی و خدمات  بهداشتی درمانی شهید صدوقی یزد می باشد.

Title: مقایسه شاخصهای مدیریت خدمات بهداشتی روستایی قبل و بعد از اجرای پزشک خانواده درمراکزبهداشتی درمانی روستایی استان یزد در سال92

http://tbj.ssu.ac.ir/article-1-1835-fa.html

چکیده مقدمه: یکی از روش های رایج برای مدیریت خدمات  بهداشتی وهدایت صحیح بیماران در نظام تأمین سلامت، استفاده از پزشک خانواده است. برنامه پزشک خانواده که قریب 8 سال درمناطق روستایی با هدف بهبودشاخص های سلامت وتقویت نظام ارجاع ازاجرای آن می گذرد، ضروری است مورد ارزیابی قرار گیرد. روش بررسی: این پژوهش، از نوع توصیفی می باشد. اطلاعات شاخص ها ی مدیریت بهداشتی ، جهت سال 1383 ونیمه اول 1384 بعنوان قبل، وسال های 1389و1390 بعنوان بعد از اجرای برنامه پزشک خانواده، در38 مرکز بهداشتی درمانی روستایی استان یزد بصورت سرشماری انجام شد. جامعه آماری این پژوهش، شامل پزشکان خانواده، ماماها وواحدهای پاراکلینکی مراکزروستایی بود.  برای جمع آوری اطلاعات، از ابزارچک لیست محقق ساخت با 15سؤال در پنچ مؤلفه(نسبت جمعیت به پزشک، نسبت ماما به مرکز، درصددسترسی به آزمایشگاه، متوسط اقلام دارویی موجود درداروخانه مراکزروستایی، ومتوسط زمان حضور  پزشک درشهرستان های استان یزد) وپس ازانجام روائی مورداستفاده قرارگرفت. جهت استخراج نتایج از نرم افزار Spssبا ویرایش 19 وآزمون های آماری Mann Whitney test & T- test استفاده گردید. یافته ها: مقایسه شاخصهای مدیریت خدمات بهداشتی، قبل و بعد از اجرای طرح پزشک خانواده، نشان داد، شاخص نسبت ماما به مرکز بطور معنی داری بهبود چشمگیری داشته است، واجرای برنامه پزشک خانواده نتوانسته برمیانگین سایرشاخص های ذکرشده با وجود بهبودنسبی تأثیرمثبت داشته باشد (05/0 p< ). نتیجه گیری: می توان با تقویت شاخص های مدیریت خدمات بهداشتی مطابق بااستانداردهای مربوطه ونیزدرنظرگرفتن امکانات رفاهی، وانگیزه های مناسب مالی، جهت افزایش زمان ماندگاری پزشکان در نقاط محروم روستایی، درآینده تقویت نظام ارجاع ودرنتیجه ارتقاء شاخص های سلامت را شاهد باشیم.
